# Trends of the World Input and Output Network of Global Trade

**DOI:** 10.1371/journal.pone.0170817

**Published:** 2017-01-26

**Authors:** Rita María del Río-Chanona, Jelena Grujić, Henrik Jeldtoft Jensen

**Affiliations:** 1 Facultad de Ciencias, Universidad Nacional Autónoma de México, Mexico City, Mexico; 2 Centre for Complexity Science and Department of Mathematics, Imperial College London, South Kensington Campus, SW7 2AZ, London, United Kingdom; Universidad Rey Juan Carlos, SPAIN

## Abstract

The international trade naturally maps onto a complex networks. Theoretical analysis of this network gives valuable insights about the global economic system. Although different economic data sets have been investigated from the network perspective, little attention has been paid to its dynamical behaviour. Here we take the World Input Output Data set, which has values of the annual transactions between 40 different countries of 35 different sectors for the period of 15 years, and infer the time interdependence between countries and sectors. As a measure of interdependence we use correlations between various time series of the network characteristics. First we form 15 primary networks for each year of the data we have, where nodes are countries and links are annual exports from one country to the other. Then we calculate the strengths (weighted degree) and PageRank of each country in each of the 15 networks for 15 different years. This leads to sets of time series and by calculating the correlations between these we form a secondary network where the links are the positive correlations between different countries or sectors. Furthermore, we also form a secondary network where the links are negative correlations in order to study the competition between countries and sectors. By analysing this secondary network we obtain a clearer picture of the mutual influences between countries. As one might expect, we find that political and geographical circumstances play an important role. However, the derived correlation network reveals surprising aspects which are hidden in the primary network. Sometimes countries which belong to the same community in the original network are found to be competitors in the secondary networks. E.g. Spain and Portugal are always in the same trade flow community, nevertheless secondary network analysis reveal that they exhibit contrary time evolution.

## Introduction

International trade is a key part of the global economy. A common approach to study international trade is to analyse input-output tables, which was developed in 1941 by Wassily Leontief [1] when he divided the economy in a number of sectors which would trade with each other. In order to rank the sectors he developed a procedure which is considered to be an early example of the PageRank measure [2], which would later obtain fame as being a crucial part of the Google’s algorithm [3]. This work brought him a Nobel Prize in Economics in 1973. With the growth of economic data availability, input-output networks have been increasingly analysed using network theory. Sectors or countries are usually considered as the nodes of the networks and links represents the transaction between them. One of the first datasets to become available was the International Trade Data [4] which contains information about the trade flow for different products for a large number of countries. The properties of the resulting network - the International Trade Network (ITN)—has been extensively investigated [5–7], observing fat-tail distributions. The data has also been used to form a so called product space, where products are linked with a proximity measure [8, 9]. The data set can also be used to construct bipartite networks of countries and their export products. This network has formed the basis of attempts to predict future economic development of specific countries [10, 11] and to define new metrics which in the case of [12] has yielded new and very important insights.

More recently, a World Input Output Dataset became publicly available [13]. The database covers 40 countries, including the world’s largest economies, and annual trade between 35 different sectors within these countries for the period from 1995 to 2009, hence the financial crash in 2008 is included. The network properties of this data set show similarities with the ITN, namely the fat-tail degree distribution [14–16]. The dataset is very helpful for the examination of the importance of different sectors using different kinds of centrality measures [15, 17]. It was also used to observe different economic trends like rise of China [16] and as a testing bed for the analysis of the influence of economic shock through analysis of the cascading failures of this network [18, 19].

To assess changes in network properties a comparison between different years was done in Ref. [20]. However the evolution of network centrality measures were never used to infer the properties of the system. Here we consider two networks. In the first, the nodes represent countries and the second the nodes represent sectors. We analyse these two networks in the same manner. Namely, for each year we compute two different network measures of each node. The first consists in the PageRank [21] of the individual nodes. The second measure is the strength, also called weighted degree, which is the sum of the weights of the links connected to a node. The result consists of two time series for each node. Next we construct secondary networks in which the nodes represents these time series. We compute the Pearson correlation coefficient between the time series. If this correlation coefficient is above a certain threshold we define the two corresponding nodes as being connected by a link. Finally, we analyse these secondary network in order to determine which countries, or sectors, form modules of strongly interdependence in the sense that nodes within a module are influencing each other more than they influence nodes outside the given module.

## Methods

The World Input Output Database (WIOD) [22] has been developed to enable analyse of the effects of globalisation on trade patterns, stress on the environment and the socio-economic development across a wide set of countries. The database covers 27 EU countries and 13 other major economies (Canada, United States, Brazil, Mexico, China, India, Japan, South Korea, Australia, Taiwan, Turkey, Indonesia, Russia) in the world. Trade with countries not among those listed is aggregated into one post labelled “trades with the rest of the world” or RoW, for the period from 1995 to 2009. Although the RoW is an artificial economy and not a country we include it in the analysis as it allows countries which major trade is not in Europe to maintain their trade information. The entire data set covers more than 85% of world GDP in 2008.

To analyse the WIOD dynamics we study the evolution of the PageRank and the strength (weighted degrees) of the nodes in the network. We define two networks: one consisting of countries (Country-WION) and one in which the nodes represent sectors (Sector-WION). The purpose of having country and sector aggregated networks is to present a clear and straight-forward analysis of the methodology that yields simple to analyse results. For each network we perform a community analysis and study the evolution of these communities. Furthermore we study the time evolution of the PageRank [21] of the nodes of the two networks.

In addition we define the strength of each node as the sum of the weights (transaction flow) of all the links and investigate the dynamics of these strengths. In this way we obtain two time series for each node in our networks (i.e. both for the country and for the sector networks). We consider countries’/sectors’ time series to be the nodes of the secondary networks (correlation networks) and links between them to indicate correlation. This is done in the following way: We compute the Pearson correlation coefficient [23], which is given by:

$$\begin{array}{c} r_{xy}=\frac{\sum x_{i}y_{i}-n\bar{x}\bar{y}}{\sqrt{\left(\sum x_{i}^{2}-n{\bar{x}}^{2}\right)}\;\sqrt{\left(\sum y_{i}^{2}-n{\bar{y}}^{2}\right)}}, \end{array}$$ (1)

where *x*_*i*_ and *y*_*i*_ denotes data points of time series *X* and *Y* respectively, each of length *n*. In our case *X* can, for example, represent the time series of PageRanks of node labelled **x** and *Y* denotes the equivalent time series of node **y**. The correlation coefficient assumes values in the interval [−1, 1].

We want correlation networks which link only the most strongly correlated and the most strongly anti-correlated nodes. Therefore we apply a thresholds *T* to the Pearson correlation coefficients (*r*_*xy*_)—we include a link between nodes **x**, **y** in the correlation networks only if |*r*_*xy*_| > |*T*|. Note that thresholds for correlation are positive and for anticorrelation negative, we denote thresholds for correlation and anticorrelation by *T*^+^ and *T*^−^ respectively. To avoid arbitrary thresholds we have designed the following iterative procedure by which we gradually filter the edges. Our method is similar to other filtering approaches which make use of the mean and standard deviation to choose which correlated pairs to include [24–26]. The procedure to define the thresholds is the following:

First, we compute the Pearson correlation coefficient between the time series of all pairs of different nodes in the network. We partition the set of these Pearson correlation coefficients into two, the positive and the negative. Then we calculate the average value of positive Pearson coefficients (${\bar{r}}^{+}$) and of negative Pearson coefficients (${\bar{r}}^{-}$) with their standard deviation (*σ*^+^, *σ*^−^). We start with initial thresholds given by averages: $T_{0}^{+}={\bar{r}}^{+}$ and $T_{0}^{-}={\bar{r}}^{-}$. After we increase/decrease the thresholds by defining $T_{n}^{\pm }=T_{n-1}^{\pm }\pm \sigma^{\pm }$. We continue defining new thresholds until thresholds *n* breaks the condition |*T*^±^| < 1.

**Fig 1:**
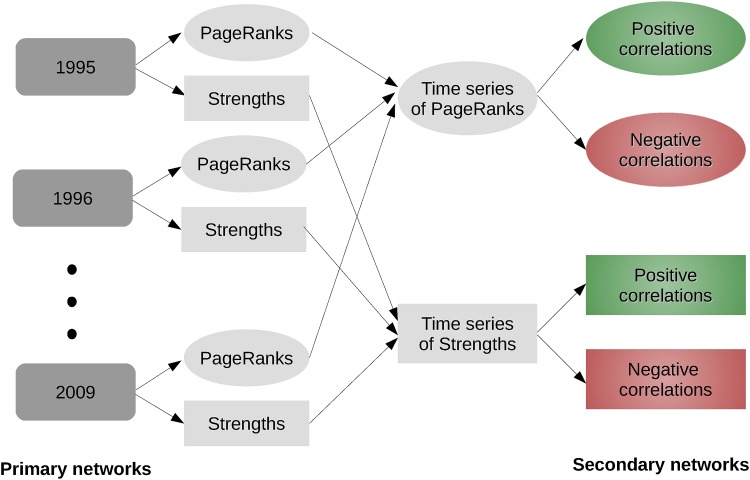
How do we form the secondary networks? We start with primary networks where nodes are countries and the links are total annual exports from one country to the other. We form 15 such networks for each year starting from 1995 until 2009. We calculate PageRanks and Strength of each country in these networks and form time series of PageRanks and Strengths for each country. Finally we calculate positive and negative correlations between these time series and form 4 different secondary networks, where nodes are countries and links are one of the following: positive correlations between PageRanks series, negative correlations between PageRanks series, positive correlations between Strengths series or negative correlations between Strengths series. We repeat the same procedure for the sectors.

We found that these derived secondary correlation networks allow a clear identification of the role of a country or sector during a given time period. In the following section we explain in detail the procedure we propose for the analysis of the dynamics of input-output networks. The diagram of the process is in Fig 1.

Let us describe how we determine the most important nodes within each network. To do this, we analyse network centralities. Here we concentrate on two. One is strength centrality which is the sum of the weights of in-degree and the weights out-degree of a node. For our network the strength is equivalent to the sum all the imports (in weight) and exports (out weight) made by a country or sector in a year. Since all countries or sectors will be influenced by the overall global trend spurious correlations between individual pairs of nodes are often induced through this shared dependence. We therefore normalise all the strengths by the total trade for that year. The other centrality measure is PageRank which is the algorithm developed and used by Google to detect the most important pages in the web, in our network it corresponds to the most important countries or sectors in a given year. Below, we briefly explain the PageRank computing procedure for a graph a graph *G* with set of links and nodes *E*(*G*), *V*(*G*).

Define:

$$\begin{array}{c} P_{1}\left(i,j\right)=\left\{ \begin{array}{cc} 1/deg^{+}\left(i\right) & :(i,j)\in E(G)\\ 0 & :\text{else} \end{array}\right. \end{array}$$

Where *i*, *j* are nodes in the graph and *deg*^+^(*i*) denotes the out-degree - which can be weighted- of node *i*. Let *n* = |*V*(*G*)| and *J*_*n*,*n*_ be the *n* × *n* matrix which entrances are all ones. Form *P*_2_ by replacing zero rows of *P*_1_ by vector $\frac{1}{n}J_{n,1}$. Now, define the PageRank Matrix by:

$$\begin{array}{c} P\left(G\right)=\left(1-c\right)P_{2}+\frac{c}{n}J_{n,n}. \end{array}$$ (2)

Where *c* is the damping factor, originally set to 0.85. The PageRank vector is given by the stationary distribution (dominant eigenvector) of the PageRank matrix. This vector is usually computed by the power method [27].

The implementation of this measurement was done with python library igraph which uses the power method. The parameters we use are the default (e.g. *c* = 0.85) but we consider the direction and weights of the links (or self-loops) [28].

Both Pagerank and strength are computed for all years for each country in the country network and for each sector in the sector network. In this way we obtain time series for each country and sector describing the time evolution of their PageRank and the economic strength. These time series are analysed and compared to important economic events such as crisis.

For each year between 1995 and 2009 we perform community detection on our networks. We use the optimal modularity algorithm [29] from the Python library igraph [28]. We present the time dependence of the communities in the results and discussion section.

## Results and Discussion

In this section we present our results in three parts. First we analyse the PageRank and normalised strength time series of the six leading countries, although we are able to obtain interesting insights this analysis is very is time consuming. Second we find communities of the Country-WION which obey geographic and political relations. In the third part we propose a method to analyse the dynamics in a more efficient manner than in the first part. Here we find that by comparing both PageRank and normalised strength networks we can observe common clusters, which we interpret as countries/sectors that are subject to similar economic dynamics in contrast to countries/sectors with essentially independent dynamics. For sectors, the correlation networks reveal clusters which are related to the specifics of the resource (renewable and non-renewable) the sectors depend on. With these finding we show that the correlation networks provide significant additional insights. Although in this work we portray simple examples to outline the methodology and its advantages, in the supporting information we show that this methodology can be applied to larger and disaggregated data sets. Its application highlight interesting features and encourage further work on the analysis of correlation networks. Furthermore in this section we also present the results of a randomized test which shows the significance of the correlations found.

For an easier discussion we use abbreviation for countries and sectors. In Tables B and A in S1 File we present the complete name and its short name.

### Phenomelogical analysis with Pagerank and Strength

#### Country-WION Analysis

**Fig 2:**
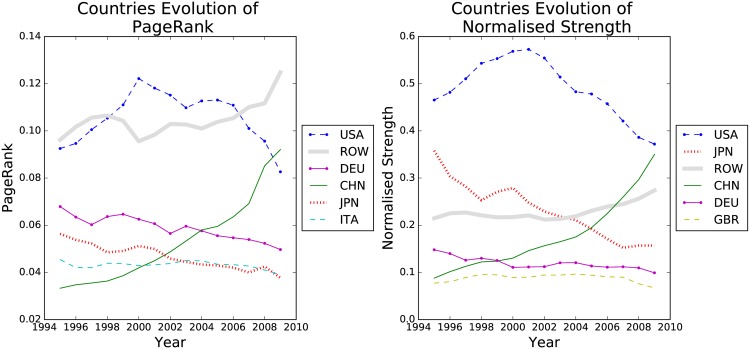
Evolution of the Country network over time. Top 6 ranked countries by Pagerank (left) and by normalized strength (right).

Countries and sectors are ranked according to the sum of the centrality measure of their corresponding node over the years 1995 to 2009. For simplicity we focus on the subset of the top six countries. Analysing the full set of time series for all countries is numerically very demanding. We do return to the full set later when analysing the correlation network. For now we focus on the six most important countries in the Country-WION network with respect to strength and PageRank centrality. We notice that the network follows a fat-tail distribution [30] and accordingly we expect the top nodes to dominate the behaviour of the network. Concerning countries of lower rank we refer the reader to supplementary material. The top six countries were the same for both centrality measures, as shown in Fig 2. However the order differs slightly, most noticeable Germany’s rank drops two places from PageRank to normalised strength, implying that Germany’s (DEU) position in the trade network makes it more important than Japan (JPN) and China (CHN), although these countries have bigger economies by total GDP. China is on a rising trajectory according to both measures from year 2000 onward while USA experience a slow but steady decrease since 2006.

Analysing each of the 40 countries’ (plus the Rest of the World, ROW) node centrality time series is time consuming and may not reveal the actual interdependencies between the nodes. In the Correlation Network section we propose a simple and efficient method to supplement the analysis.

#### Sector-WION Analysis

**Fig 3:**
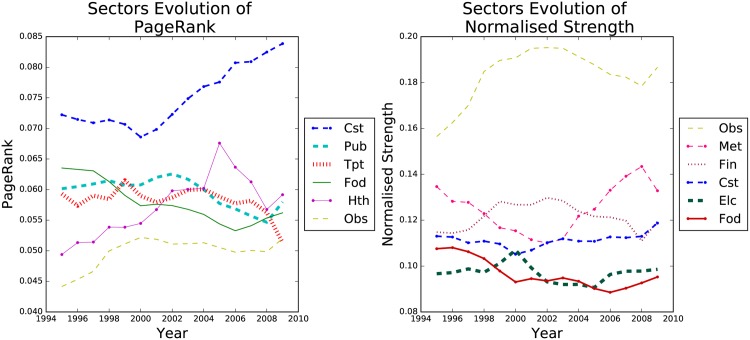
Evolution of sectors over time. Top 6 ranked sectors by pagerank (left) and strength (right).

The same analysis was done for sectors shown in Fig 3. Here PageRank and Strength identify different sectors for the top 6 rank, only “Renting of M&Eq and Other Business Activities” (Obs), Construction (Cst) and Food, Beverages and Tobacco (Fod) sectors are ranked among the top 6 in both. Meaning that the relation between importance in the network and economy size is stronger for countries than for sectors. We notice that Construction (Cst), is roughly fourth largest sector by strength, but by PageRank it is clearly the most central sector. Also construction centrality increases form the year 2000 onwards. All these suggests that Construction is the most influential sector, even though it was never the largest one. This helps explains why the crises that started in the Housing market, which is part of the the Construct sector, had such a devastating effect on world economy in 2008. When it collapsed, although it was not the largest market, it had a strong impact in the economy due to its high centrality. PageRank analysis of more recent dataset could give us early warnings of which markets are fundamental for the stability of the economy.

### Communities

**Fig 4:**
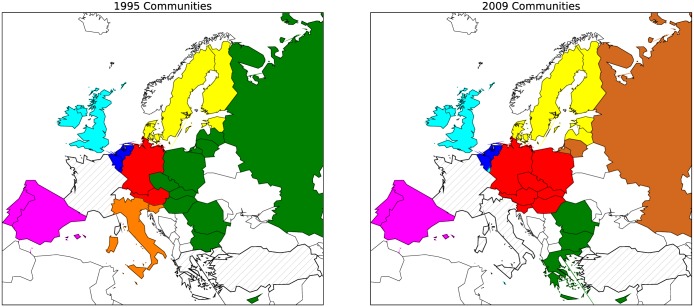
1995 and 2009 Country-WION community structure. In this map countries of the same community are of the same color, with the exception of white countries (non-coloured countries), which are those that are not in the World Input-Output Dataset and dashed countries which are on the dataset but make a community on their own. It can be observed that the communities in this network are mostly geographical and how the countries of the former Eastern Block changed their trade from Russia to Germany.

The optimal modularity algorithm implemented with igraph [31] [28] revealed communities of the Country-WION for all years. We found communities of more than one country only in Europe, all other countries in the dataset make a community of their own. In Fig 4 we show the obtained communities in Europe for the year 1995 and 2009. The communities we found can be explained by geographical proximity and historical connections: Portugal and Spain make one community, UK and Ireland, Benelux countries etc. But we also notice changes in the community structure which are consequences of the political changes: large part of the Eastern European countries which once belonged the Eastern block, belong to the same economic community as Russia in 1995. However large number of those countries changed their trade towards Germany and by 2009 many belong to a community which includes Germany. However, as will be shown in the following section, countries of the same community are not necessarily subject to similar dynamics in the network. Therefore belonging to the same community does not necessary mean that the two economies influence each other, and could be that one country is just a market for the other stronger economy.

For the Sector-WION the optimal modularity algorithm revealed no community structure. This result is natural, as sectors are divisions of the market, defined to have the least overlap. Furthermore, this actually confirms that the sectors used in the World Input-Output Table are well defined.

### The Correlation Network

Below we make reference the United Nations Regional Groups, which are geopolitical regions. Particularly we will be referring to the Eastern European Group (EEG) and the Western European and Others Group (WEOG). Regarding the thresholds computed we note that for all the networks considered $|T_{3}^{\pm }|>1$, few presented $|T_{2}^{\pm }|<1$ furthermore no network had more than two links with weights over *T*_2_. Therefore only thresholds *T*±_0_ and *T*±_1_ are used to create the correlation/anticorrelation networks. The links above these thresholds will be coloured light or dark gray respectively.

#### Country-WION, PageRank

**Fig 5:**
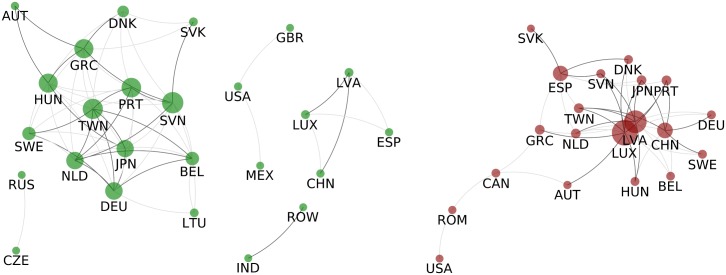
Correlations of Countries’ PageRank. Left: Correlated countries - Two countries are connected with a dark grey link if the Pearson coefficient between their PageRank time series is above $T_{1}^{+}=0.872$ if the coefficient is only above $T_{0}^{+}=0.78$ the link is light grey. Right: Anticorrelated countries - Two countries are connected in dark grey if their PageRank time series have a Pearson coefficient below $T_{1}^{-}=-0.88$ if the coefficient is only below $T_{0}^{-}=-0.79$ the link is light grey. In both the size of of the nodes is proportional to the strength of the node (regarding light edges).

The positive correlation network (Fig 5 left) may give insight to regional and political cooperation of countries, for example USA is linked to Mexico (MEX) and Great Britain (GBR). These correlations are expected, USA-Mexico for geographic reasons and USA-UK for historical ones, yet in the primary network they never belong to the same community. Then, we see that economies of Russia and Czech Republic are also correlated even if in the primary networks by 2009 they no longer belong to the same community. India is correlated with the Rest of the World (RoW) countries, again expected since most of India’s neighbours are not part of the dataset and are represented only as a part of RoW. However the major connected group is not purely geopolitical, it is composed of 13 countries: 8 members of the WEOG (AUT, BEL, DEU, DNK, GRC, NLD, PRT and SWE; 3 of the EEU (HUN, SVK and SVN) and 2 Asian (JPN and TWN). Finally, we have the puzzling cluster of China, Latvia (LTV), Luxembourg (LUX) and Spain (ESP), which is difficult to explain with regional cooperation (only LUX and ESP are in the same regional group), political relationships or historical reasons. However, the correlation might not be due to pure coincidence. If we look at the PageRank series of CHN, LUX, LVA and ESP (Fig F in S1 File) we note a growing PageRank for most of the period 1995-2009 in all of these four countries, so the correlation could be due to common cause. This cause could be, for example, that the large economic growth of the world in the period studied influenced these four economies the most. Unfortunately after 2008 crises the growth in Spain and Latvia stopped. However to study these phenomenon in a more profound manner other data sets are needed.

The anti-correlation network shown in Fig 5 (left) indicates that the dynamics of CHN, LUX, LVA and ESP strongly differ from the rest of network. LUX and LVA link to the largest group of positively correlated countries (AUT, DNK, SVK, GRC, HUN, SVN, PRT, SWE, DEU, NLD, BEL, JPN and TWN);

The growth of Chinese economy is a common knowledge at this point however, it is not clear which markets is China overtaking. China strongly anticorrelates with Germany, Taiwan, Sweden, Japan and Portugal. Furthermore CHN’s PageRank is comparable to these countries’ PageRank - specially to DEU’s and JPN’s - suggesting these are the countries China overtook. On the contrary, although LUX and LVA link to several countries (e.g. DEU, HUN, JPN, NLD, etc.), their PageRank (Fig F in S1 File) is negligible in comparison to these large group of countries, therefore we cannot assume a considerable competition effect between them. Another prominent anticorrelation is between Spain and Portugal. The two countries are always in the same module in the primary network, however the secondary networks suggests that large amount of trade between two countries does not necessary means that two economies will be correlated, on contrary we see that on the example of Portugal and Spain the opposite is happening.

For a deeper analysis of the anticorrelations mentioned above, the anticorrelation between the sectors of these countries must be explored. Although in this work we do not focus on these deeper analysis we show an example with the SWE’s and CHN’s strongly anticorrelating sectors. We can see that the industries which might have been overtaken by the Chinese economy are Agriculture, Food, Textiles, Paper & Printing, Rubbers & and Water and Air Transport (see Fig E in S1 File). The anticorrelations between the transport sectors can be explained by the development of the Chines shipping industry which started in that period, and probably took part of the Swedish market.

#### Country-WION, Normalised Strength

**Fig 6:**
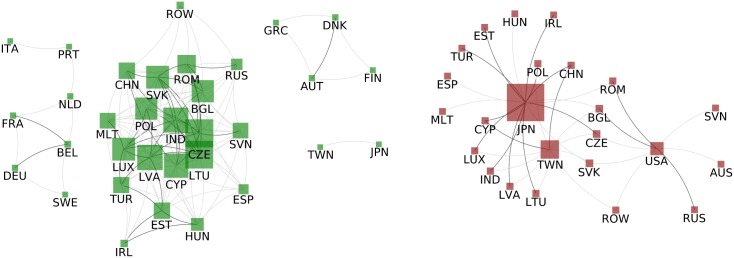
Countries Normalised Strength correlation. Left: Correlated countries - Two countries are connected with a dark grey link if the Pearson coefficient between their strengths time series is above $T_{1}^{+}=0.92$ if the coefficient is only above $T_{0}^{+}=0.823$ the link is light grey. Right: Anticorrelated countries - Two countries are connected in dark grey if their strengths time series have a Pearson coefficient below $T_{1}^{-}=-0.84$ if the coefficient is only below $T_{0}^{-}=-0.75$ the link is light grey. In both the size of of the nodes is proportional to the strength of the node (regarding light edges).

We proceed to analyse the correlations between the normalised strength (Fig 6). The positive part (left) presents 4 connected groups. The biggest is composed by 19 countries: 11 members of the EEU (BGL, CZE, EST, HUN, LTU, LVA, POL, ROM, RUS, SVN, SVK), 5 members of the WEOG (ESP, IRL, LUX, MLT, TUR) and other 3 countries/group of country (CHN, IND/ROW). Other two connected groups are formed only by members of the WEOG (AUT, DNK, FIN and GRC / BEL, FRA, DEU, ITA, NLD, PRT and SWE). The last connected group is of Asian countries (JPN and TWN). Interestingly WEOG members are correlated both according to PageRank and Normalised Strength, while EEU members are mostly only correlated according to strength. This implies that WEOG members effort to gain presence in the network coincide with the importance in the Network. A possible reason can be mutual cooperation, since normalised strength is associated to imports and exports amount and PageRank relates to importance. We stress that although CHN, LUX, LVA and ESP were correlated PageRank wise, the absence of correlation in normalised strength indicates that they obey different economical policies. In this manner, the correlation network for PageRank and normalised strength provide insight of cooperation in the network that could not be easily obtained with only the time series plot.

In the network of negative correlations for normalised strength (Fig 6) JPN, TWN and USA stand out. The two Asian countries link to countries of the biggest component of the respective positive correlation network. JPN and CHN PageRank are comparable, regarding pure exports/imports they are in direct competition. USA strongly links to EEG members (RUS, ROM and BGL) history may explain the competition among them. Here the negative correlation network could suggest competing countries, both directly in exports/imports by normalised strength and also for the significance in the network as indicated by PageRank.

#### Sector-WION, PageRank

**Fig 7:**
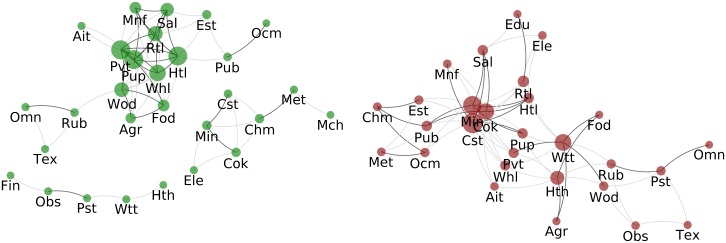
Correlation of Sectors’ Pagerank. Left: Correlated sectors - Two sectors are connected with a dark grey link if the Pearson coefficient between their PageRank time series is above $T_{1}^{+}=0.88$ if the coefficient is only above $T_{0}^{+}=0.79$ the link is light grey. Right: Anticorrelated countries - Two sectors are connected in dark grey if their PageRank time series have a Pearson coefficient below $T_{1}^{-}=-0.87$ if the coefficient is only below $T_{0}^{-}=-0.79$ the link is light grey. In both the size of of the nodes is proportional to the strength of the node (regarding light edges).

The positive correlation network for PageRank of sectors (Fig 7 left) the biggest connected group is composed mostly by sectors related to renewable resources (Agr, Fod, Wod and Pup) or related to public services (Htl, Pvt, Rtl, Sal and Whl) while the second largest group is composed by sectors related to non-renewable resources (Min, Cok, Cst, Chm, Met and Mch). In this form the correlation network suggests that the importance of a sector in the network depends considerably on the type of resource of use (considering that public services primary “resource” are employers).

The right side of Fig 7 presents the anti-correlation network. Cok, Cst and Min have the most links, from Fig 3 and Fig H in S1 File we observe that their PageRank rapidly increased, implying that they have risen in importance in the network while several other sectors decreased theirs.

#### Sector-WION, Normalised Strength

**Fig 8:**
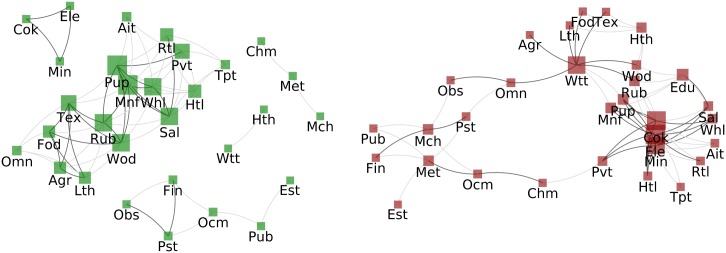
Correlation of Sectors’ Normalised Strength. Left: Correlated sectors - sectors are connected with a dark grey link if the Pearson coefficient between their strengths time series is above $T_{1}^{+}=0.91$ if the coefficient is only above $T_{0}^{+}=0.82$ the link is light grey. Right: Anticorrelated sectors - Two sectors are connected in dark grey if their strengths time series have a Pearson coefficient below $T_{1}^{-}=-0.89$ if the coefficient is only below $T_{0}^{-}=-0.80$ the link is light grey. In both the size of of the nodes is proportional to the strength of the node (regarding light edges).

The positive correlation network (Fig 8 (left)) again reveals a major connected group of sectors that utilise renewable resources (Agr, Fod, Lth, Pup, Tex and Wod) another consist of public services (Htl, Pvt, Rtl Sal, Tpt and Whl) and finally another connected group is formed by sectors with non-renewable resources (Cok, Min and Ele).

The right side, which describes the anti-correlation between the sectors’ normalised strengths, is similar to the PageRank network in Fig 7 in the sense that nodes Cok, Ele and Min which are related to non-renewable resources have many links. For sectors, the main difference between PageRank and normalised strength correlation networks is that sectors related to renewable resources are positively related in both networks while sectors related to non-renewable resources correlate mostly in the PageRank network.

We note that our methodology can also be implemented to larger data sets. An example of this can be seen on the supporting information.

## Conclusions

We analysed the World Input Output Network with a focus on the dynamics of the importance of nodes (countries or sectors) as measured by either PageRank or economical strength. We find the PageRank and strength allows for complimentary insights. First we analyse the primary networks of countries or sectors where the weight of the links are given by the annual trade flow. The correlations over time between nodes are used to construct secondary networks which contains information about the similarity of the development of the two given countries or sectors. Furthermore, we construct networks based on the negative correlations, which pin-points the countries or sectors that are in competition with each other. We find that these secondary networks gives us a new valuable information. For example Portugal and Spain are always in the same module when we analyse the primary network, which is understandable given their geographical proximity and historical connections. However, when we analyse the secondary network we find that they are actually negatively correlated with each other, suggesting that these country are in competition with each other. Furthermore, we identify a similarity of behaviour of Latvia, Luxembourg and China and to some extend Spain, however this behaviour is unlikely a consequence of a real connection of these countries. This shows that we, obviously, have to be careful when interpreting correlations. However the investigation of the correlation network can be a very powerful tool, which help to identify possible interesting dynamics and can suggest where additional analysis is needed in order to test potential interrelationships. Furthermore, our approach may help clarify possible connections between different economies or even reveal anticorrelation as in the case of Spain and Portugal. The rise of China is very well documented by now, however our analysis is able to suggest the markets China is overtaking; namely the markets of Germany, Japan as well as Taiwan, Portugal and Sweden. We find evidence of three large “local” leaders, which are Germany, USA and China whose development is strongly correlated with the surrounding countries. This is another aspect which can not see from the primary network. We find that sectors separate into two different groups, sector based on renewable resource and the once based on non-renewable resources. However in general the sectors are found to anti-correlate which may suggest highly competitive relationships. Obviously correlations do not necessarily imply causation, it will therefore be of great interest in the future when sufficient data becomes available to do the above network analysis using information theoretic causality measures. Our work encourages further research regarding the topology of the correlation and anticorrelation networks, which could contribute to the analysis of these networks. Finally, this method can be expanded to be used on any case where we have temporal networks.

## Supporting Information

S1 File Supporting Information. This file contains additional figures and text which expand the results and address the statistical validity of them.
(PDF)
