# Supplementary material for: Trends of the World Input and Output Network of Global Trade
Source: PLoS One. 2017 Jan 26;12(1):e0170817. doi: 10.1371/journal.pone.0170817 (PMC5270329; doi:10.1371/journal.pone.0170817)
Supplement: S1 File — This file contains additional figures and text which expand the results and address the statistical validity of them. (PDF) [file pone.0170817.s001.pdf]

Supporting Information for the paper Trends of the World Input and Output Network of Global Trade

Rita María del Río-Chanona[1], Jelena Grujić[2, ¤, *] Henrik Jeldtoft Jensen[2]

1 Facultad de Ciencias, Universidad Nacional Autónoma de México, Mexico City, Mexico
2 Centre for Complexity Science and Department of Mathematics, Imperial College London, South Kensington Campus, SW7 2AZ, UK

¤Current Address: AI-lab, Computer Science Department, Vrije Universiteit Brussel, Brussels, Belgium & MLG group, MLG, Département d'Informatique, Université Libre de Bruxelles, Brussels, Belgium
*** jgrujic@vub.ac.be**

The meaning of the abbreviations for countries and sectors used along the article can be found on table A and table B. In this section we show further information regarding the dynamics of the World Input Output Network. We present correlation networks for the disaggregated World Input Output Network and present a test of the statistical validity of our method. Furthermore we highlight the similar PageRank dynamic of China, Spain, Luxembourg and Latvia in Fig F. Also we present the PageRank series of the 40 countries and 35 sectors in the network in Figs G and H.

Regarding statistical validity It could be argued that correlation between the node's time series is a casualty or false positive. That is to say, if we keep the links of the network but distribute them in a random fashion we might still detect several correlations between these randomized time series. In order to disprove this hypothesis we conduct a randomized test.

In this test, we preserve important feature about an input-output network. The network is dense and directed but more importantly the edge's weight $w_{u,v}$ is related to the edge's weight $w_{v,u}$ (the import value is similar to the export value). Secondly there are strong self-loops in each node. To keep this two key-characteristics we shuffle the pairs of in-out links and reassigning them to other pairs of nodes, that is to say if link with weight $w_{u,v}$ is randomly reassigned to edge (x, y) then weight $w_{v,u}$ is automatically assigned to edge (y, x). This way every pair of nodes is reassigned strictly one weighted out-link and one weighted in-link which are related just as in the original network. Likewise we shuffle the self-loops and reassign them to a different (or same) node, every node gets one self-loop, none is left with out one.

With these new randomized networks we implement our methodology that is we calculate the Pearson correlation coefficients for the nodes' time series, threshold them as explained above and form the correlation networks. This randomized procedure was done 1000 times, after we analyse overall statistical properties of the semi-random correlation networks, namely mean number of correlations above the zero-threshol (T_0) and standard deviation.

In table C we present the results of the random simulation: mean and standard deviation of the number of strong correlation found. Here we also present the strong correlations found in the networks we study. It can be observed that the number of strong correlations we found are much more than the ones found in the semi-random input-output networks. Therefore showing the statistical validity of our methodology.

Table A. Sectors abbreviations

Abbreviation	Sector
Agr	Agriculture, Hunting, Forestry and Fishing
Ait	Air Transport
Chm	Chemicals and Chemical Products
Cok	Coke, Refined Petroleum and Nuclear Fuel
Cst	Construction
Edu	Education
Elc	Electrical and Optical Equipment
Ele	Electricity, Gas and Water Supply
Est	Real Estate Activities
Fin	Financial Intermediation
Fod	Food, Beverages and Tobacco
Hth	Health and Social Work
Htl	Hotels and Restaurants
Ldt	Inland Transport
Lth	Leather and Footwear
Mch	Machinery, Nec
Met	Basic Metals and Fabricated Metal
Min	Mining and Quarrying
Mnf	Manufacturing, Nec; Recycling
Obs	Renting of M&Eq and Other Business Activities
Ocm	Other Community, Social and Personal Services
Omn	Other Non-Metallic Mineral
Otr	Other Supporting and Auxiliary Transport Activities; Activities of Travel Agencies
Pst	Post and Telecommunications
Pub	Public Admin and Defence; Compulsory Social Security
Pup	Pulp, Paper, Paper , Printing and Publishing
Pvt	Private Households with Employed Persons
Rtl	Retail Trade, Except of Motor Vehicles and Motorcycles; Repair of Household Goods
Rub	Rubber and Plastics
Sal	Sale, Maintenance and Repair of Motor Vehicles and Motorcycles; Retail Sale of Fuel
Tex	Textiles and Textile Products
Tpt	Transport Equipment
Whl	Wholesale Trade and Commission Trade, Except of Motor Vehicles and Motorcycles
Wod	Wood and Products of Wood and Cork
Wtt	Water Transport

Table B. Countries abbreviations

Abbreviation	Country
AUT	Austria
AUS	Australia
BEL	Belgium
BGL	Bulgaria
BRA	Brazil
CAN	Canada
CHN	China
CYP	Cyprus
CZE	Czech Republic
DEU	Germany
DNK	Denmark
ESP	Spain
EST	Estonia
FIN	Finland
FRA	France
GBR	Great Britain
GRC	Greece
HUN	Hungary
IDN	Indonesia
IND	India
IRL	Ireland
ITA	Italy
JPN	Japan
KOR	Korea
LTU	Lithuania
LUX	Luxembourg
LVA	Latvia
MEX	Mexico
MLT	Malta
NLD	Netherlands
POL	Poland
PRT	Portugal
ROM	Romania
RUS	Russia
SVK	Slovakia
SVN	Slovenia
SWE	Sweden
TUR	Turkey
TWN	Taiwan
USA	United Stated of America
ROW	Rest of the World

Correlation Networks for bigger systems So far we implemented the method on aggregated data sets (by country and by sector), we did this to have clear and simple results which would facilitate the understanding and advantages of it. However this method can be implemented to broader data sets, for example the whole World Input Output Networks with 1435 nodes. Here we illustrate a brief implementation of the method to the PageRank series of this bigger data set.

After computing the PageRank series of the 1435 nodes for all the years $(1995-2009)$, we calculate the thresholds as explained in the methodology section and obtain three valid thresholds for the positive correlations $T_0^+ = 0.78$, $T_1^+ = 0.87$, $T_2^+ = 0.96$. We investigate only the links above the last one (T_2^+). Furthermore we only present blocks (biconnected subgraphs) of the correlation network with at least 10 nodes, with this restriction only 3 blocks were found which are shown in Figs A, B and C.

The first block of the correlation network, shown in Fig A, is dense and composed almost entirely by the manufacturing sector (Mnf). Which might suggest that manufacturing is an activity performed by countries in conjunction or that manufacturing is an activity governed by global trends and therefore many countries' manufacturing sectors are correlated. The second block (Fig. B) is not as densely connected at the first, however at the centre several nodes of Canada's sectors can be seen densely connected, therefore it seems that we are actually observing the trends of

Table C. Random simulation results for the correlation and anticorrelation networks

**Network**	Mean number of strong correlations	Standard deviation of number of strong correlations	Strong correlations in our networks
Countries' PageRank correlation	4.87	0.15	62
Countries' PageRank anticorrelation	2.28	1.58	44
Countries' strength correlation	22.79	4.11	141
Countries' strength anticorrelation	0.03	0.19	36
Sectors' PageRank correlation	3.54	1.84	62
Sectors' PageRank anticorrelation	1.55	1.39	65
Sectors' strength correlation	15.60	3.24	92
Sectors' strength anticorrelation	0.02	0.16	36

Canada's sectors and sectors of other countries it relates to. Furthermore this finding suggests Canada has the most independent economy, in terms of correlation mostly between the country itself. Of course this suggestion might be biased to the dataset used and must therefore be taken with caution. Finally the third block (Fig. C) includes many different countries and sectors, which makes a straight forward analysis difficult. The use of centrality measures and community detection algorithms is encouraged to be used when analysing such networks.

The anticorrelation network also presented three valid thresholds: $T_0^- = -0.77$, $T_1^- = -0.85$, $T_2^- = -0.94$. We present the results highest threshold (T_2^-), here only one block of more than 10 nodes was formed, which is shown in Fig D . The number of nodes in this block is remarkable and makes simple analysis difficult. As in the case of correlation networks the use of centrality measures and community detection can be used, however the interpretation of results must be done carefully. The topology of correlation and anticorrelation networks must be considered, for example it is likely that correlation networks have a high clustering coefficient (number of triangles) while the anticorrelation network may present few or no odd-cycles.

To have a deeper analysis of the relationships between countries suggested in this work, one could focus on the correlation of countries' sectors. In Fig. E we present an example of these countries' most anticorrelated sectors (above T_3^- of the anticorrelation network obtained from the disaggregated data set).

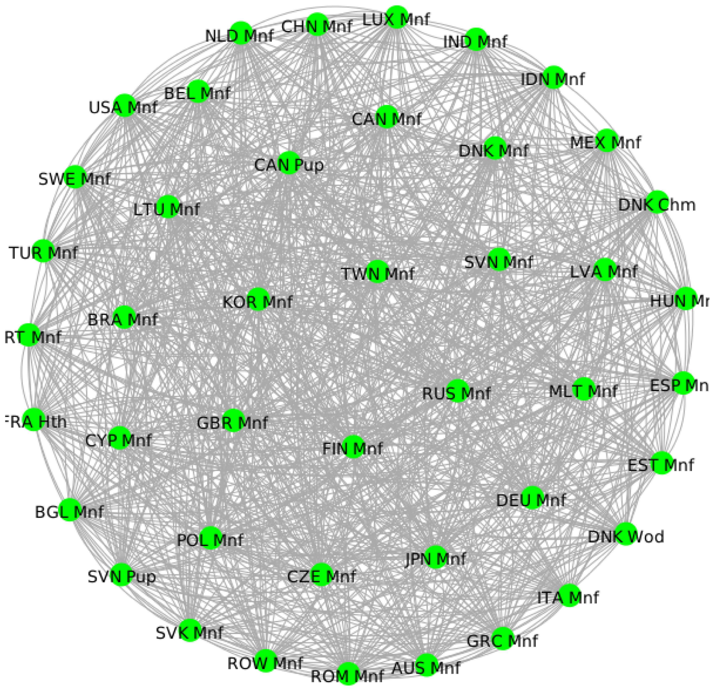

Fig A. Highly correlated block of nodes in the WION, manufacturing sectors of several countries are densely correlated

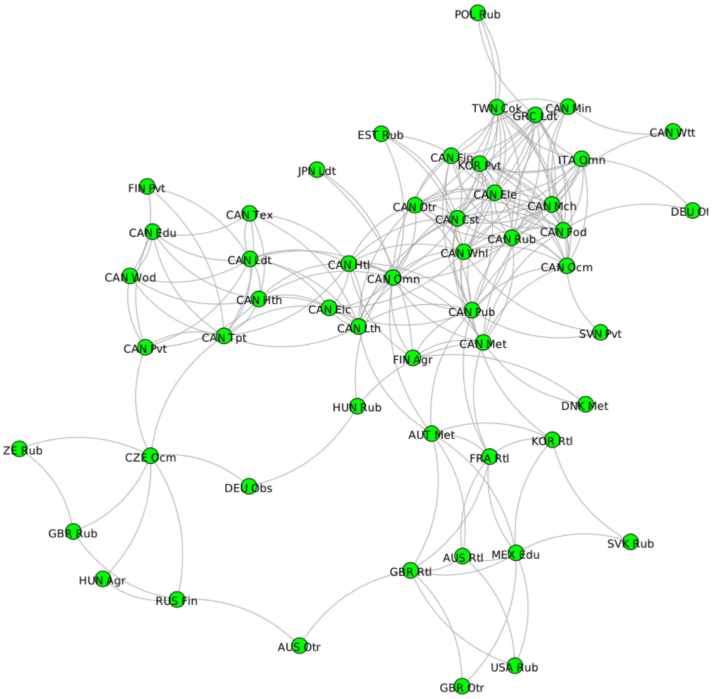

Fig B. Highly correlated block of nodes in the WION, Canadian sectors are densely correlated

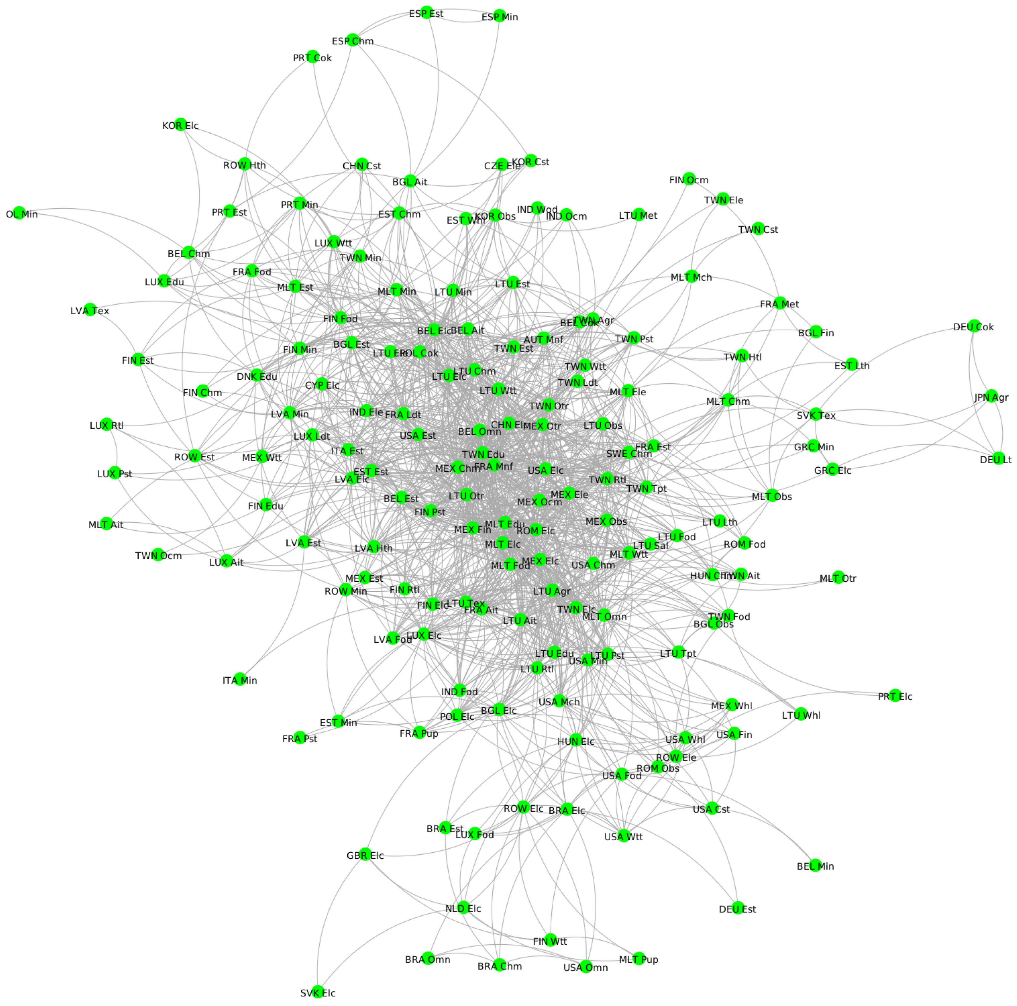

Fig C. Highly correlated block of nodes in the WION

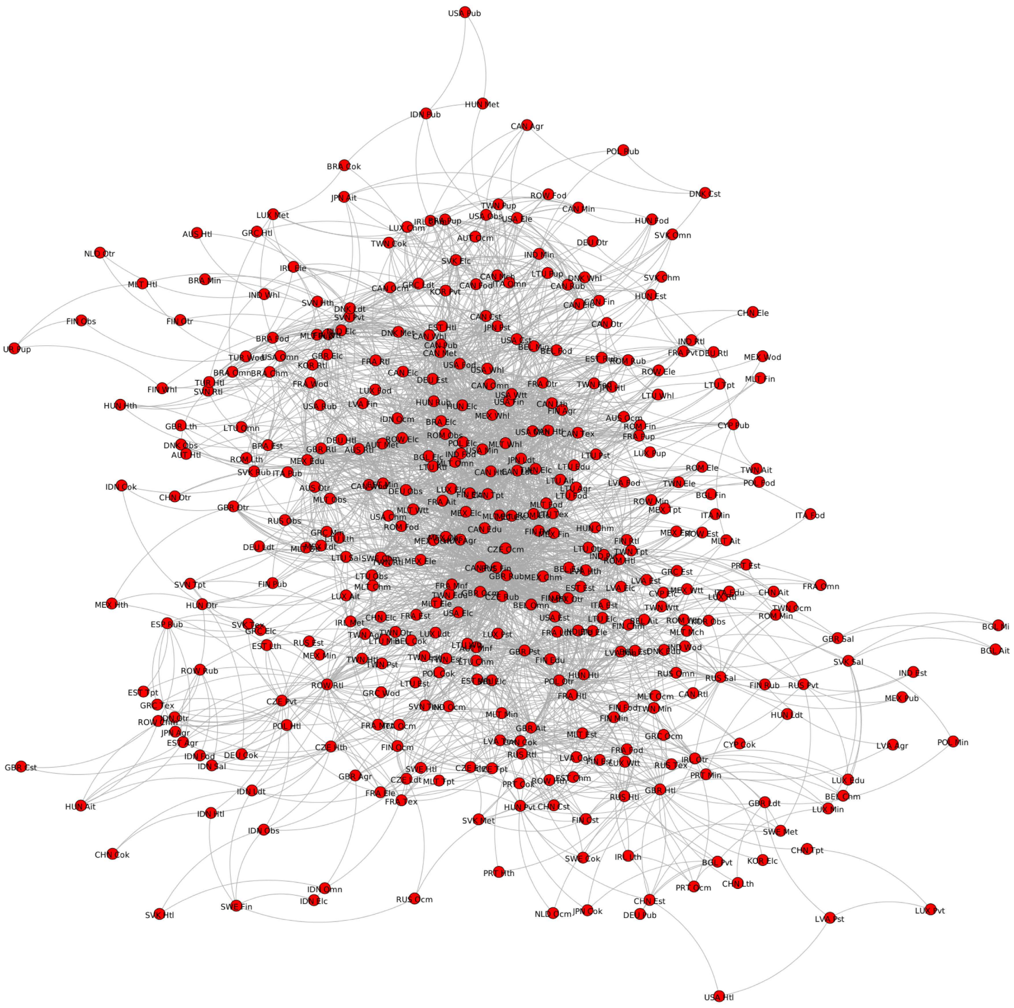

Fig D. Highly anticorrelated block of nodes in the WION

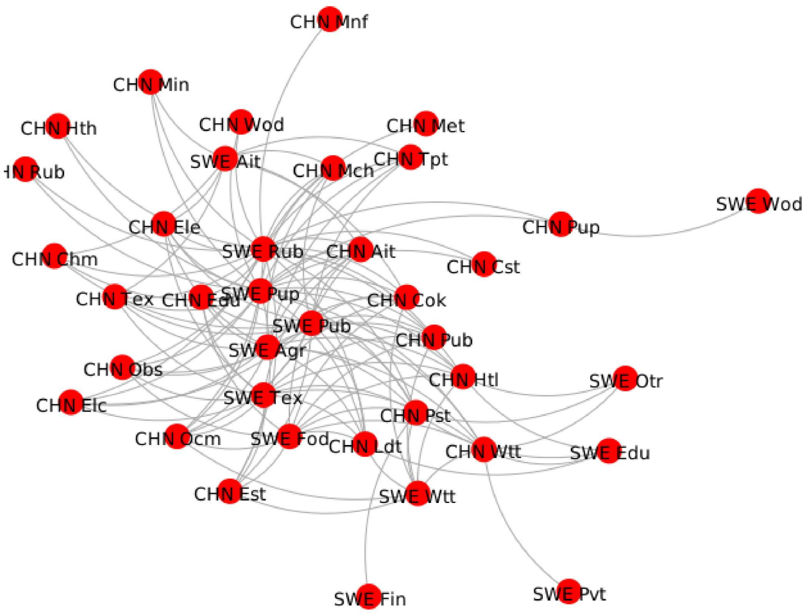

Fig E. Anticorrelation between Chinese and Swedish sectors. The industries overtaken by the Chinese economy are mainly: Food, Agriculture, Textiles, Paper & Printing, Rubers & Platicas and Air and Water Transport.

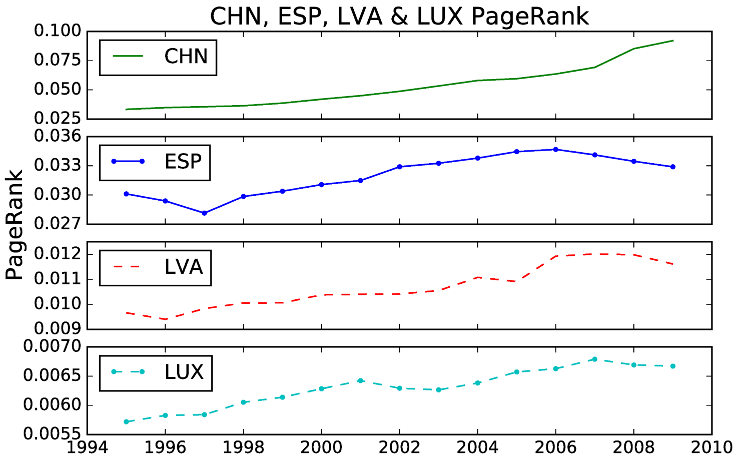

Fig F. CHN, ESP, LVA & LUX. Countries which PageRank increases.

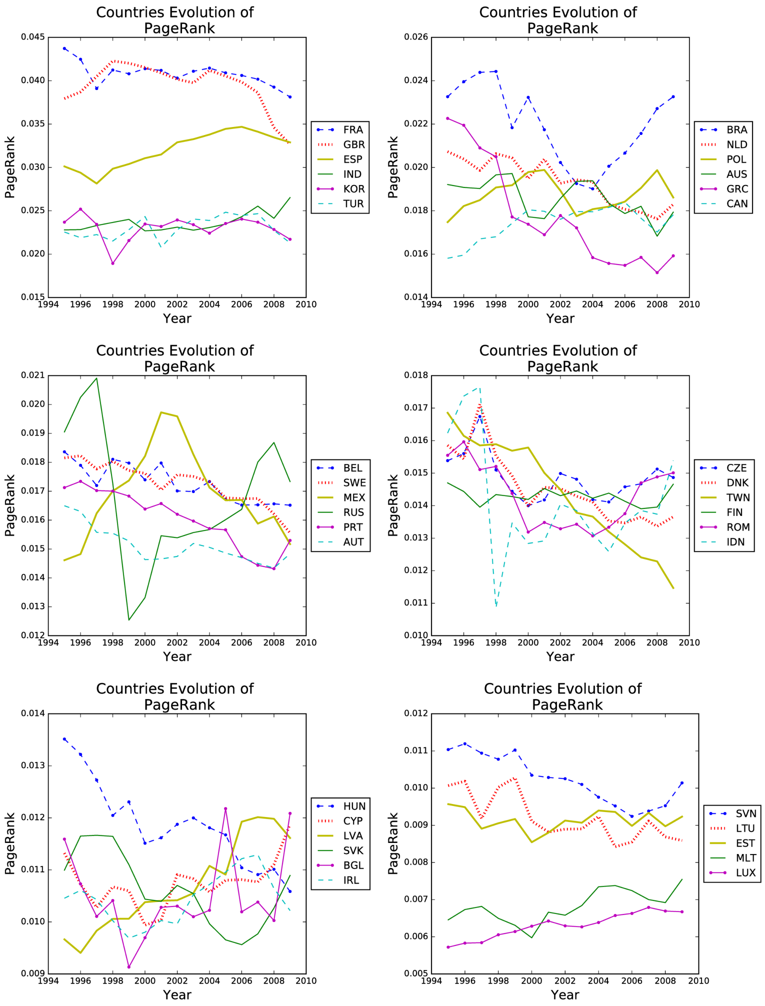

Fig G. Countries PageRank evolution ranked below top 6. Here we include the PageRank time series for the countries in the WION that were not shown elsewhere

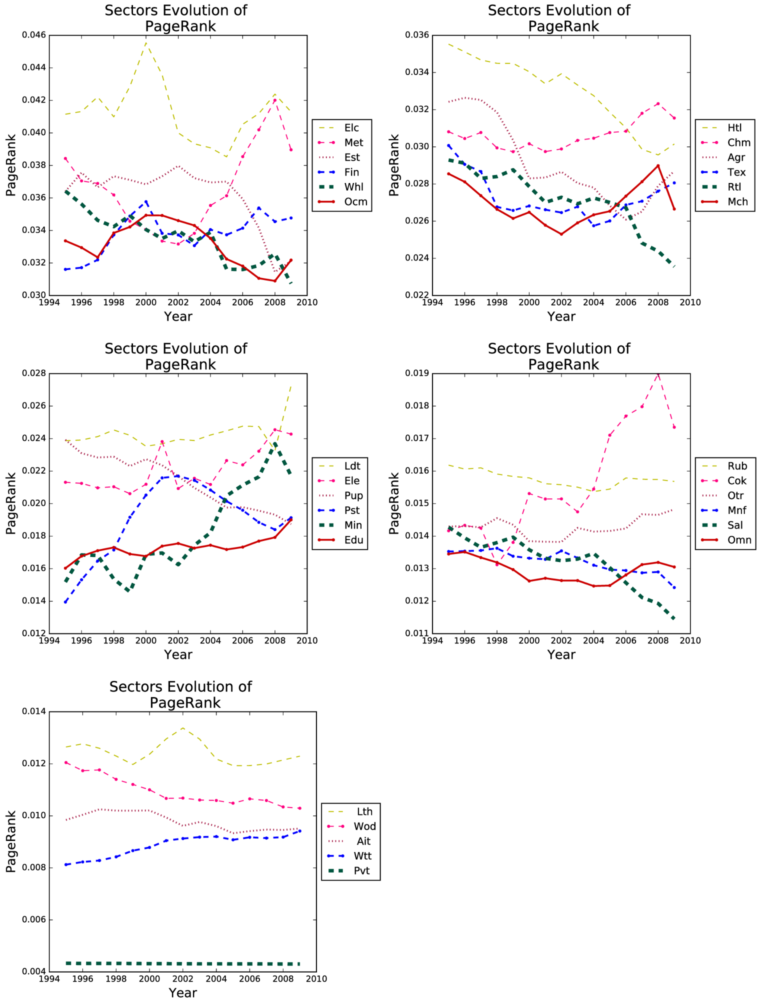

Fig H. Sectors' PageRank evolution ranked below top 6. Here we include the PageRank time series for the sectors in the WION that were not shown elsewhere

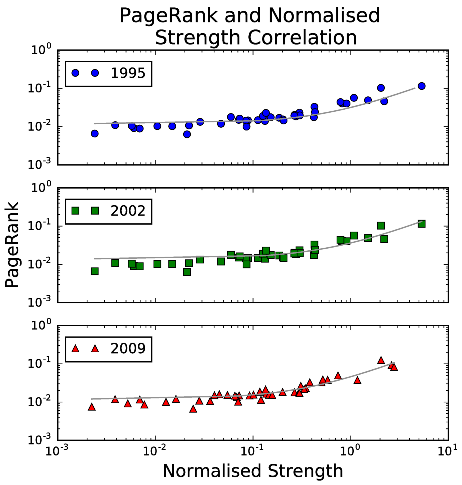

Fig I. PageRank vs Strength (left). Linear fit for years $1995, 2002$ and 2009 are shown. The fit parameters are as follows: $m_{1995} = 0.019$, $m_{2002} = 0.022$, $m_{2009} = 0.031$; $b_{1995} = 0.015$, $b_{2002} = 0.014$, $b_{2009} = 0.012$; $r_{1995} = 0.87$, $r_{2002} = 0.91$, $r_{2009} = 0.92$; $p_{1995} = 8.5 \times 10^{-14}$, $p_{2002} = 1.5 \times 10^{-16}$, $p_{2009} = 5.6 \times 10^{-18}$
